# Chemotherapy Alters the Phylogenetic Molecular Ecological Networks of Intestinal Microbial Communities

**DOI:** 10.3389/fmicb.2019.01008

**Published:** 2019-05-07

**Authors:** Jing Cong, Jingjuan Zhu, Chuantao Zhang, Tianjun Li, Kewei Liu, Dong Liu, Na Zhou, Man Jiang, Helei Hou, Xiaochun Zhang

**Affiliations:** ^1^Department of Medical Oncology, The Affiliated Hospital of Qingdao University, Qingdao University, Qingdao, China; ^2^Cancer Institute, Qingdao, China

**Keywords:** intestinal microbiota, phylogenetic molecular ecological networks, high-throughput sequencing, chemotherapy, colorectal cancer

## Abstract

Intestinal microbiota is now widely known to play key roles in nutritional uptake, metabolism, and regulation of human immune responses. There are multiple studies assessing intestinal microbiota changes in response to chemotherapy. In this study, microbial phylogenetic molecular ecological networks (pMENs) were firstly used to study the effects of chemotherapy on the intestinal microbiota of colorectal cancer (CRC) patients. Based on the random network model, we demonstrated that overall network structures and properties were significantly changed by chemotherapy, especially in average path length, average clustering coefficient, average harmonic geodesic distance and modularity (*P* < 0.05). The taxa in the module tended to co-exclude rather than co-occur in CRC patient networks, indicating probably competition relationships. The co-exclude correlations were decreased by 37.3% from T0 to T5 in response to chemotherapy. Significantly negative correlations were observed in positive/negative OTU degree and tumor markers (*P* < 0.05). Furthermore, the topological roles of the OTUs (module hubs and connectors) were changed with the chemotherapy. For example, the OTU167, OTU8, and OTU9 from the genera *Fusobacterium, Bacteroides*, and *Faecalibacterium*, respectively, were identified as keystone taxa, which were defined as either “hubs” or OTUs with highest connectivity in the network. These OTUs were significantly correlated with tumor markers (*P* < 0.05), suggesting that they probably were influenced by chemotherapy. The pMENs constructed in this study predicted the potential effects of chemotherapy on intestinal microbial community co-occurrence interactions. The changes may have an effect on the therapeutic effects. However, larger clinical samples are required to identify the conclusion.

## Introduction

Colorectal cancer (CRC) is a major killer of people around the world despite continuing medical advances on several fronts. Chemotherapy continues to be the mainstay therapy for most CRC patients, and the related treatment response is unpredictable. Personalized cancer therapies are now emerging, and targeted therapies have promoted revolutionary outcomes in CRC (Mirnezami et al., 2012). However, novel problems such as idiosyncratic adverse effects, acquired resistance and high costs are still present (Kantarjian et al., 2013; Welsh et al., 2016). Recent studies have implicated intestinal microbiota at the species level in influencing the drug response and toxicity of CRC patients (Haiser et al., 2013). Drug metabolism by intestinal microbiota has been well recognized since the 1960s (Scheline, 1968). Intestinal microbiota plays a key role in confirming the efficacy and toxicity of a broad range of drugs (Li et al., 2016). With the development of high-throughput sequencing, the importance of intestinal microbiota for drug modulation and discovery is increasingly recognized (Jia et al., 2008). Scott et al. (2017) reported that intestinal microbiota influenced fluoropyrimidines, which are the first-line treatment for CRC through drug interconversion involving bacterial vitamin B6 and B9 and ribonucleotide metabolism. Guthrie et al. (2017) suggested that metagenomic mining of the microbiome, which is associated with metabolomics, was considered as a non-invasive approach to develop biomarkers for CRC treatment outcomes. As a consequence, intestinal microbiota can be leveraged for therapeutic interventions (Ross et al., 2016) and to improve therapeutic effects (Wallace et al., 2010).

Microbes coexist in complex environments in which interactions among individuals are indispensable for community assembly and ecosystem function (Fuhrman, 2009; Hallam and Mccutcheon, 2015). However, few studies have explored the interactions among intestinal microbiota or determined which individuals share niches within the intestinal environment. Therefore, identifying and defining the interactions that occur among intestinal microbiota contributes to understanding the role of intestinal microbiota to provide a possibility for better therapeutic interventions. Molecular ecological network analysis provides a promising future for exploring the dynamics of microbial interactions and niches (Faust and Raes, 2012). In recent years, molecular ecological network analysis has been used as a tool to determine complex microbial assemblages in various environments such as in humans (Faust et al., 2012), groundwater (Deng et al., 2016), and soil (Jing et al., 2015). Network analysis can identify putative keystone taxa that are important for maintaining community structure and function (Power et al., 1996).

To identify intestinal microbial assemblages that potentially interact with or share niches within the intestine, we constructed the phylogenetic molecular ecological networks (pMENs) for healthy volunteers and CRC patients during the chemotherapy based on the 16S rRNA sequencing data (Deng et al., 2012). We studied intestinal microbiota using fecal samples from CRC patients and healthy volunteers. High-throughput sequencing of 16S rRNA gene amplicons was used to describe the intestinal bacterial assemblages. We mainly examined the changes in intestinal microbiota during the treatment based on the pMENs. The following research questions were addressed based on the intestinal microbial phylogenetic data: (i) whether chemotherapy affects the intestinal microbial molecular network structure, (ii) what the potential “keystone taxa” in the network are and how they change in response to chemotherapy, and (iii) what the relationships are among key OTUs in the network and clinical variables. Our work identifies a previously undocumented dimension of intestinal microbiota and offers insights into the species-species interaction networks of intestinal microbiota associated with CRC patients in response to chemotherapy.

## Materials and Methods

### Experiment Description

Twenty-two CRC patients from the Affiliated Hospital of Qingdao University (Qingdao, China), aged 34–73 years, were enrolled in our study (Table 1). This study was selected based on the histopathological diagnosis of primary CRC, newly diagnosed and untreated, and no history of other tumors. Twenty-one healthy volunteers, aged 27–64 years, were selected as controls. During a routine physical examination, all the participants who had used antibiotics within the past 2 months or were regularly using non-steroidal anti-inflammatory drugs, probiotics, or statins before sampling, were excluded from the study. Other exclusions included chronic bowel disease, food allergies, dietary restrictions, and other signs of infections. Based on the national comprehensive cancer network (NCCN) guidelines, the CRC patients were treated with standard chemotherapy, and follow-up samples were obtained from CRC patients before the treatment of every stage. The interval time between two treatments is about 21 ± 3 days. However, individuals had different starting time at the first chemotherapy. The sampling time followed the treatment time for about six times. In total, 123 fecal samples from the CRC patients and 21 fecal samples from healthy individuals were collected. The demographic, clinical and technical details of study subjects were shown in Table 1 and Supplementary Table S1. All these participants had been local residents of Qingdao city for more than 5 years. This study was approved by the Ethics Committee of the Affiliated Hospital of Qingdao University and all study participants gave written informed consent before participation. These fresh fecal samples from healthy individuals were collected into 5 ml tubes in the morning at home. Then they were sent into our lab within 3 h after defecation, and were reserved by ice bag during the transportation, and were immediately frozen at -80°C. These samples from CRC patients were collected in the morning in the hospital, then they were immediately frozen at -80°C after defecation until the day of analysis. The collected samples from the healthy individuals and CRC patients were named by H and T, respectively. In addition, these samples from CRC patients before the first chemotherapy, before the second chemotherapy, before the third chemotherapy, before the fourth chemotherapy, and before the fifth chemotherapy were named by T0, T1, T2, T3, T4, and T5, respectively.

**Table 1:** Summary information of individuals in this study (Fecal sample’s demographic, clinical, and technical details).

Group	Healthy volunteers	Colorectal cancers patients	*P* value	Sample collection	Sequencing platform
Sample size	21	22	NA	Prior to each treatment	Sequencing Platform: Illumina Hiseq PE250; Sequencing Target Depth: 5 GB; read length: 500 bp
Male/Female	4/17	14/8	0.003		
Age, year, median	54 (26–64)	57 (34–73)	0.098		
BMI, median	23.2	23.4	0.913		

### DNA Extraction, Purification, Sequencing, and Data Processing

Microbial DNA was extracted from fecal samples with the QIAamp Fast DNA Stool Mini Kit as previously reported (Wu et al., 2016). The freshly extracted DNA was purified by 1% melting point agarose gel followed by phenol chloroform-butanol extraction. The V3-V4 region of the 16S rRNA gene from each sample was amplified by the bacterial universal primers (forward primer, 5′-ACTCCTACGGGRSGCAGCAG-3′, and reverse primer, 5′-GGACTACVVGGGTATCTAATC-3′). PCR amplification was performed in a 30 μl reaction containing 15 μl 2 × KAPA HiFi Hotstart ReadyMix, 10 ng template DNA, 1 μl of each primer (forward and reverse primers), and ddH_2_O. The reaction mixtures were amplified using the following conditions: denaturation at 95°C for 1 min; 12 cycles of 98°C for 15 s, 72°C for 10 s, 94°C for 20 s, 65°C for 10 s, and 72°C for 10 s; 11 cycles of 94°C for 20 s, 58°C for 30 s, and 72°C for 30 s; and a final extension at 72°C for 150 s. The PCR amplification products were purified using an AxyPrep DNA Gel Extraction Kit (Axygen, United States), eluted in 30 μl water, and aliquoted into three PCR tubes. DNA quality and quantity were assessed by the 260/280 and 260/230 nm ratios, and the final DNA contents were quantified with a Qubit^®^ dsDNA HS Assay Kit (Invitrogen, United States). Finally, we sequenced the bacterial DNA amplicons from each fecal sample using an Illumina Hiseq 2500.

Raw sequences were separated into samples by barcodes. After raw data quality control, the clean reads were first sorted by abundance from large to small, followed by the removal of singletons. Using the Usearch software, the reads were clustered and chimeras were removed based on 97% standard similarity. The reads from each sample were randomly pumped to extract the corresponding Operational Taxonomic Unit (OTU) sequences. Each OTU is considered to represent a species. The taxonomic assignment was performed by the ribosomal database project (RDP) classifier (Wang et al., 2007). Random resampling was conducted on 39,796 sequences per fecal sample.

### Network Construction and Analysis

In this study, we took 22 CRC patients and 21 healthy individuals as biological replicates to infer the possible co-occurrence relationship between intestinal microbes in response to chemotherapy. Networks were constructed for intestinal microbiota based on the OTU relative abundance. Each stage of chemotherapy was considered as one treatment/group. Only OTUs detected in more than 50% of replicate samples in each group were used for network construction. The microbial phylogenetic data were converted to a similarity matrix. The similarity matrix measures the degree of concordance between the abundance of OTUs across various samples by obtaining the absolute values of the Pearson correlation matrix (Horvath and Dong, 2008). The microbial communities can be predicted by the Poisson distribution, which reflects the non-random properties, system-specific of a complex system, and the Gaussian orthogonal ensemble (GOE) statistics, which is involved in the random properties of a complex system (Luo et al., 2007). The random matrix theory (RMT), which is a reliable, sensitive and robust tool for analyzing high-throughput genomics data (Deng et al., 2012), was used to automatically determine the appropriate similarity threshold (St) for identifying modular networks and elucidating network interactions. The St represents the minimal strength of the connections between each pair of nodes (Zhou et al., 2011). Subsequently, an adjacency matrix was derived from the similarity matrix based on the St (Luo et al., 2007). These transitions are indicated by the existence of the same St (Table 2). Global network properties were characterized according to previous research (Deng et al., 2012). Modules reflect divergent selection regimes, clusters of phylogenetically and closely related species, habitat heterogeneity and the key unit of species co-evolution (Olesen et al., 2007). Modularity is used to demonstrate a network that is naturally divided into distinct sub-groups (Deng et al., 2012). The average degree represents the complexity of the network. Harmonic geodesic distance represents the state (close or dispersive) of the nodes in the network. The average clustering coefficient describes how well a node is linked to its neighbors. The modular topological roles are explained by peripherals, connectors, module hubs and network hubs, which are defined by two parameters, the within-module connectivity (*Zi*) and among-module connectivity (*Pi*) (Deng et al., 2012; Jing et al., 2015). The *Zi* reflects how close a node is connected to other nodes within its own module, and *Pi* describes how close a node contacts with different modules. Module hubs have the nodes with *Pi* ≤ 0.62 and *Zi* > 2.5, connectors with *Pi* > 0.62 and *Zi* ≤ 2.5, and network hubs with *Pi* > 0.62 and *Zi* > 2.5, peripheral nodes with *Pi* ≤ 0.62 and *Zi* ≤ 2.5. In an ecological perspective, peripherals tend to have the role of specialists, whereas connectors and module hubs are considered as generalists and network hubs as super-generalists (Olesen et al., 2007).

**Table 2:** Topological properties of the empirical molecular ecological networks (MENs) of intestinal microbial community from healthy individuals and colorectal cancer (CRC) patients during the different treatment stages and their associated random MENs.

Sample		H	T0	T1	T2	T3	T4	T5
Empirical Network	No. of original OTU^a^	655	778	727	793	814	788	758
	Network size^b^	105	99	102	103	80	103	103
	Total links	110	238	228	239	173	210	231
	Average degree	2.095	4.808	4.471	4.641	4.325	4.078	4.485
	Average path distance	5.942^d^	2.893^d^	2.982^d^	3.216^d^	3.403^d^	3.701^d^	3.210^d^
	Average clustering coefficient	0.07^e^	0.191^e^	0.209^e^	0.175^e^	0.140^e^	0.119^e^	0.240^e^
	Average harmonic geodesic distance	4.165^f^	2.488^f^	2.539^f^	2.759^f^	2.717^f^	3.022^f^	2.668^f^
	Identical threshold	0.660	0.660	0.660	0.660	0.660	0.660	0.660
	R^2^ of power-law	0.786	0.794	0.724	0.821	0.808	0.861	0.870
	Modularity	0.773^g^	0.429^g^	0.450^g^	0.475^g^	0.405^g^	0.500^g^	0.498^g^
Random networks^c^	Average path distance	5.584 ± 0.467	2.693 ± 0.045	2.705 ± 0.047	3.02 ± 0.052	2.883 ± 0.068	3.228 ± 0.069	3.022 ± 0.064
	Average clustering coefficient	0.014 ± 0.010	0.209 ± 0.025	0.200 ± 0.024	0.101 ± 0.018	0.139 ± 0.022	0.077 ± 0.017	0.102 ± 0.018
	Average harmonic geodesic distance	4.318 ± 0.254	2.401 ± 0.026	2.415 ± 0.026	2.635 ± 0.031	2.509 ± 0.042	2.803 ± 0.043	2.647 ± 0.039
	Modularity	0.731 ± 0.015	0.355 ± 0.010	0.381 ± 0.010	0.393 ± 0.011	0.380 ± 0.011	0.434 ± 0.012	0.396 ± 0.011

*^∗^Random networks were generated by resetting all of the links of a matching empirical network with the same nodes and links. Data were generated from 100 random runs and SD indicates the standard deviation from the 100 runs. ^*a*^Numbers of OTUs that originally used for network construction using the RMT-based approach. ^*b*^Number of OTUs (i.e., nodes) in a network. ^*c*^The random networks were generated by rewiring all of the links of a MEN with the identical numbers of nodes and links to the corresponding empirical MEN. ^*d*^Significant difference (*P* < 0.05) in average path distance between healthy individuals and CRC patients based on the Students *t*-test with standard deviations derived from corresponding random networks. ^*e*^Significant difference (*P* < 0.05) in average clustering coefficients between healthy individuals and CRC patients based on the Students *t*-test with standard deviations derived from corresponding random networks. ^*f*^Significant difference (*P* < 0.05) in average harmonic geodesic distance between healthy individuals and CRC patients based on the Students *t*-test with standard deviations derived from corresponding random networks. ^*g*^Significant difference (*P* < 0.05) in modularity between healthy individuals and CRC patients based on the Students *t*-test with standard deviations derived from corresponding random networks.*

In the RMT-based molecular ecological network approach, the phylogenetic molecular ecological networks constructed should be ensured that the co-occurrence patterns are statistically significant rather than a random process (Zhou et al., 2011). Only one network was constructed by combining 21/22 samples under each group; therefore, we cannot compare the network indices between CRC patients with chemotherapy and healthy individuals statistically. Thus, random networks were generated to assess the significance of network indices based on a null model (Sergei and Kim, 2002). Total of 100 random networks were generated in each identified network, and all network indices were calculated individually. The average and standard deviation value of all random networks were obtained (Zhou et al., 2011). The network indices between CRC patients and healthy individuals were compared based on the Student *t*-test with standard deviations derived from corresponding random networks.

### Statistical Analyses

All network analyses were performed based on the Molecular Ecological Network Analyses (MENA) Pipeline^1^ and networks were graphed using the Cytoscape 2.8.3 (Killcoyne et al., 2009) and Gephi 0.9.1 software (Eck and Waltman, 2014). The Pearson and Spearson correlation by IBM SPSS statistic 19.0 were used to determine the significance of the differences and the clinical correlates.

## Results

### Effects of Chemotherapy on pMENs

The pMENs were constructed for CRC patients in response to different therapy stages to determine the effect of chemotherapy on intestinal microbial community (Table 2). The CRC patients had unique ecological network models that varied during different treatment stages (Supplementary Figure S1). Identical threshold values of 0.66 were imposed on the T and H samples. The network sizes, links, connectivity, and module numbers were calculated for intestinal microbial community in the CRC patients and healthy individuals. Random networks were generated to test the statistical significance of the constructed network indices (Table 2). All the constructed networks showed scale-free characteristics, which means that only a few nodes in the network have many connections, whereas most nodes have no or few connections (Barabási, 2009), as proven by the power law R^2^ ranging from 0.72 to 0.87 (Table 2).

The intestinal microbial networks were significantly different for CRC patients and healthy individuals, which were proven by multiple network topological properties, such as average path length, average clustering coefficient, average harmonic geodesic distance, and modularity (*P* < 0.05, Table 2 and Supplementary Figure S1). Lower modularity values (0.40–0.50) in the T samples indicated that these networks could be impossibly isolated into multiple modules. Healthy assemblages formed larger networks with more nodes than the CRC patient networks (Table 2). However, the CRC patient networks contained more links between nodes than healthy individual networks, which increased the density of the connections and created more intricate network patterns (Supplementary Figure S1 and Table 2). The increased complexity of the T networks was also reflected by the shorter harmonic geodesic distances and the increased average degree (Deng et al., 2012). In addition, the CRC patient networks presented distinctly different from T0 to T5, identified by the changes in multiple network indices (Table 2). The average path length, average clustering coefficient, average harmonic geodesic distance and modularity, were significantly different between T0 and T5 samples (*P* < 0.05). As the treatment stages progressed, the nodes in the CRC patients increased, ranging from 99 to 103, except for T3 (80). Collectively, the above results indicated that other than the significant differences with the healthy individual networks, overall network structures of the intestinal microbial communities in CRC patients were significantly changed under the chemotherapy.

### Modularity in CRC Intestinal Communities

**FIGURE 1:**
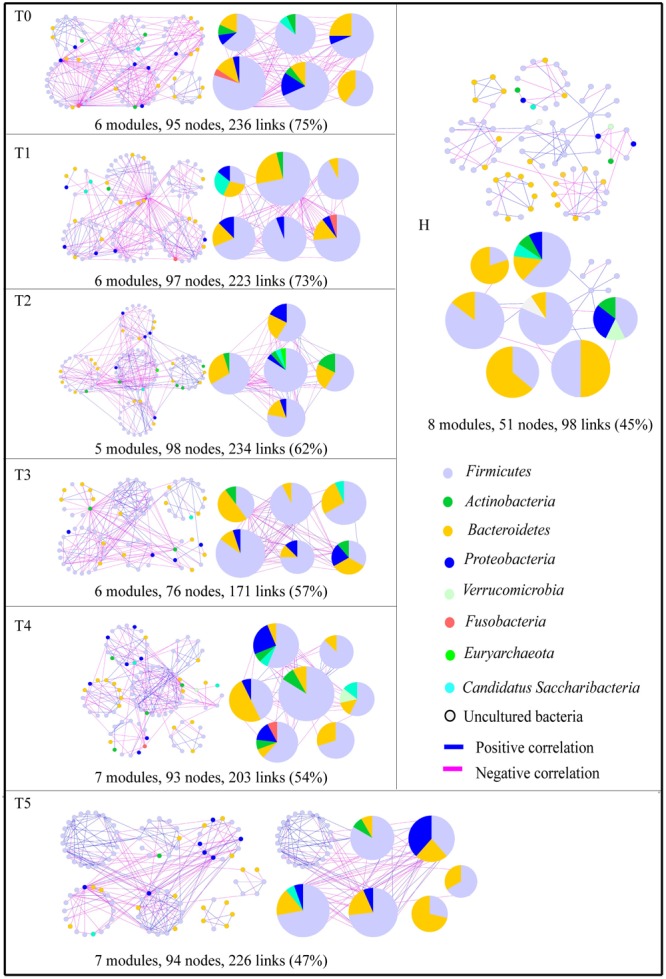
Highly connected modules within intestinal networks of colorectal cancer patients in response to the five stages of chemotherapy. Node colors represent different major phyla; pie charts represent the composition of the modules with >1 phyla. A blue link indicates a positive relationship between two individual nodes, whereas a pink link indicates a negative relationship. The number in bracket means the ratio of negative links accounting for the total links.

**FIGURE 2:**
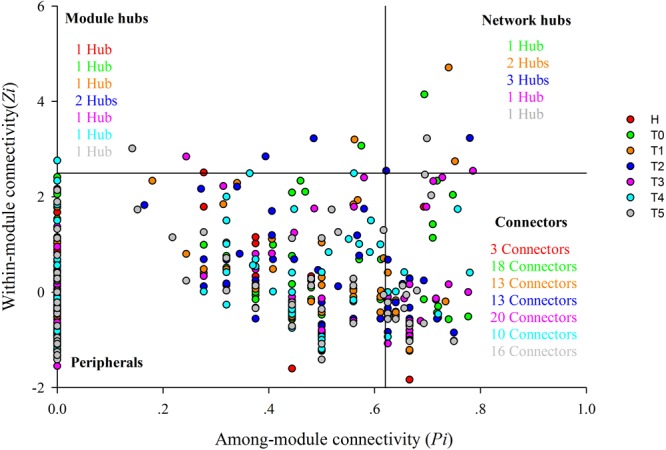
Z-P plot showing the classification of nodes to identify putative keystone species in healthy individuals and colorectal cancer patients in response to chemotherapy. Each symbol represents an OTU. Different colors represent different groups, namely, red for healthy individuals (H), green for CRC patients before the first treatment (T0), orange for CRC patients before the second treatment (T1), blue for CRC patients before the third treatment (T2), pink for CRC patients before the fourth treatment (T3), water blue for CRC patients before the fifth treatment (T4), and gray for CRC patients before the sixth treatment (T5). Module hubs have the nodes with *Pi* ≤ 0.62 and *Zi* > 2.5, connectors with *Pi* > 0.62 and *Zi* ≤ 2.5, and network hubs with *Pi* > 0.62 and *Zi* > 2.5, peripheral nodes with *Pi* ≤ 0.62 and *Zi* ≤ 2.5. There are 1, 1, 1, 2, 1, 1, and 1 module hub in H, T0, T1, T2, T3, T4, and T5, respectively. There are 1, 2, 3, 1, and 1 network hub in T0, T1, T2, T3, and T5, respectively. There are 3, 18, 13, 13, 20, 10, and 16 connectors in H, T0, T1, T2, T3, T4, and T5, respectively.

To identify microbial assemblages that potentially share or interact with intestinal niches during the CRC patient treatment process, we focused on representative networks from CRC patients with five treatment stages. We focused on modules with at least five nodes and visualized the phylogeny for these modules (Figure 1). The intestinal microbiota in CRC patients formed more complex networks than healthy individuals, as determined by more kinds of phyla in each sub-module (Figure 1). Eight modules were detected in the healthy individuals. There were 6, 6, 5, 6, 7, and 7 modules in the T0, T1, T2, T3, T4, and T5 networks, respectively (Supplementary Table S2). Networks from all CRC patients contained modules with modularity values ≤0.50 (Table 2). Overall, taxa tended to co-exclude (negative correlations, pink lines) rather than co-occur (positive correlations, blue lines); negative correlations accounted for 47–75% of the potential interactions observed at each treatment stage (Figure 2). The negative correlations in CRC patients decreased by 37.3% from T0 to T5; however, they were still more than those in healthy individuals (45%).

The composition of the modules differed within each network and changed over the treatment time (Figure 1). *Firmicutes* almost dominated all the modules from each treatment stage in CRC patients. The phylum *Fusobacteria* presented in the modules before the treatment (T0), before the second treatment (T1), and before the fifth treatment (T4). The phylum *Fusobacteria*, which was supposed to be more relevant to dysbiosis and CRC progression (Geng et al., 2014; Flemer et al., 2017), was primarily co-excluded with *Firmicutes, Bacteroidetes*, and *Proteobacteria* in the module during the different treatment stage.

### Visualization of Topological Roles of Individual Nodes in CRC Patients

To evaluate the possible topological roles of the taxa in the networks, we organized these nodes into four categories based on the among-module connectivity (*Pi*) and within-module connectivity (*Zi*) values, including the module hubs, connectors, peripherals, and network hubs (Deng et al., 2012) (Figure 2 and Supplementary Tables S3–S5). Structurally, the loss of peripherals could influence the functions of ecological networks, while losing the connectors, module hubs or network hubs would result in the deterioration of the entire network (Roger and Nunes Amaral, 2005). The nodes in each network were mainly peripherals, with most of their links inside their modules. Module hubs and connectors have been proposed to be keystone taxa because of their important roles in the network topology (Deng et al., 2012). In this study, the module hubs and connectors were detected in all networks. There were one or two nodes in each treatment stage classified as module hubs in the T networks. The module hubs identified in the T networks originated from a variety of taxonomic groups. The module hubs in T0 and T1 were from the genera *Klebsiella* and *Fusobacterium*, respectively (Supplementary Table S3), which are pathogens routinely found in the human intestine that cause diarrhea and bloodstream infections and markedly increase the rates of treatment failure and death (Yan et al., 2017). Two taxa from the genus *Bacteroides* and *Atopobium* were classified as module hubs in T2. The module hubs in T3, T4, and T5 were from *Faecalibacterium, Bacteroides*, and *Faecalibacterium*, respectively (Supplementary Table S3). Compared with the H network, the T networks had more connectors during the different treatment stages. Most of the connectors in each network originated from *Firmicutes*, while the others were from the *Actinobacteria, Bacteroidetes, Proteobacteria*, and *Candidatus Saccharibacteria* (Supplementary Table S4). In addition, there were also network hubs in the T networks, however, not in the H network. Therefore, the topological roles of the taxa were different and the keystone taxa were changed for CRC patients during the chemotherapy.

### Association of Network Structure With Clinical Variables

Spearman correlation analysis was performed between OTU degree and tumor markers (CA242, CEA, CA199, and CA724) to determine the relationships between microbial network interactions and clinical variables (Supplementary Material 1). The OTU degree was calculated by summing the strengths of the links of each node with all of the other connected nodes in the network. OTU degree represents how strong an OTU is connected to other OTUs, which is one of the most commonly used network indices (Deng et al., 2012). Significantly negative correlations (*P* < 0.05) were observed in positive/negative OTU degree and CA199 and CEA (Table 3). This means that changes of interactive relationships of pMENs between species were probably closely correlated with tumor markers in CRC patients. In addition, based on the topological roles of individual nodes in the pMENs of CRC patients, we also selected some key nodes (OTU787, OTU167, OTU8, OTU238, OTU9, OTU2, OTU847, OTU488, and OTU16) and tumor markers (CA242, CEA, CA199, and CA724) for Spearman correlation analysis to further explore the linkages between the microbial correlation networks and clinical chemotherapeutic effects. The results showed that the relative abundances of OTU167, OTU8, and OTU9 were significantly correlated with CEA, CA724, and CA242, respectively (*P* < 0.05, Supplementary Table S6). The OTU167, OTU8, and OTU9 were from the genera *Fusobacterium, Bacteroides*, and *Faecalibacterium*, respectively. These significant OTUs, which were classified into module hubs or network hubs in T0, T1, T2, and T3, were conspicuous in ecological networks. These results showed that microbial interaction networks were closely related with tumor markers, indicating that they were extremely influenced by chemotherapy. However, the relative abundance of these keystone taxa were almost not significantly correlated with chemotherapy stages (Supplementary Figure S2).

**Table 3:** The Spearman correlation of tumor markers and OTU degree.

Tumor marker	OTU degree (r, *P*)	Negative OTU degree (r, *P*)	Positive OTU links (r, *P*)
CA242	0.09, 0.87	0.31, 0.54	–0.43, 0.40
CEA	0.49, 0.33	**0.83, 0.04**	**-0.94, 0.01**
CA199	0.37, 0.47	**0.89, 0.02**	**-0.94, 0.01**
CA724	–0.03, 0.96	0.09, 0.87	–0.31, 0.54

*“r” means the Spearman’s correlation coefficient; “*P*” means the significant results. CA242, carbohydrae antigen 242; CEA, carcino-embryonic antigen; CA199, carbohydrae antigen 199; CA724, carbohydrae antigen 724. The bold values means the significant correlation between two parameters.*

## Discussion

Chemotherapy remains the mainstay treatment for patients with CRC, except for available surgical debulking (Yu et al., 2017). In recent years, the changes that occur to the intestinal microbiota in response to cancer chemotherapy have been gradually understood (van Vliet et al., 2009; Jutta et al., 2011; Stringer et al., 2013; Marin et al., 2014; Cammarota and Ianiro, 2017). Chemotherapy can damage the mucus layer and disrupt the mucosal barrier in the intestine. Afterward, some intestinal microbiota probably penetrate the lamina propria, leading to life-threatening systemic infections that activate the innate immune system, which may influence the efficacy of chemotherapy (Marin et al., 2014; Cammarota and Ianiro, 2017). A previous study reported that chemotherapy severely influenced the homeostasis of intestinal microbiota (Montassier et al., 2015); in turn, intestinal microbiota modulated the efficacy and toxicity through key mechanisms, such as the translocation, immunomodulation, metabolism, reduced diversity, and ecological variation (Alexander et al., 2017). Previous studies mainly analyzed the composition and structure of intestinal microbiota influenced by chemotherapy. Here, we firstly explored the intestinal microbial interactive networks from the feces of CRC patients in response to the five stages of chemotherapy based on the molecular ecological network analysis.

Microbes in the intestine are not independent individuals but complex inter-connected ecological communities. Microbes compete for resources or exchange genetic material, influencing the microbial composition and host health. Therefore, understanding microbial interactions is important to understand their functions. Although the chemotherapy is known to exert profound effects on intestinal microbiota (Emmanuel et al., 2014), the effects on the microbial ecological networks were first explored in this study. In our study, network properties significantly changed in CRC patients in response to chemotherapy (*P* < 0.05). For example, connectivity (average degree), which shows that how strong a node is connected to other nodes, was decreased by 6.7% in T5 than in T0. However, these CRC patients were still increased by 56.4, 53.1, 54.9, 51.6, 48.6, and 58.6% in T0, T1, T2, T3, T4, and T5, respectively, compared with healthy individuals (Table 1). It suggested that intestinal microbiota were linked with each other very tightly in CRC patients with chemotherapy. Modularity, which provides information on the extent to which nodes possess more links with their own modules, was higher by 16.1% in T5 than in T0. However, these CRC patients were lower by 44.5, 41.8, 38.6, 47.6, 35.3, and 35.6% in T0, T1, T2, T3, T4, and T5, respectively, compared with H (Table 1). These indicated that the pMENs in CRC patients were changed greatly under the chemotherapy. Previous study found that the changes of the connectivity and modularity were consistent (Liang et al., 2016). In our study, the inconsistency between the connectivity and modularity probably revealed that overall intestinal micro-ecosystem was in a seriously unbalanced state. Findings revealed that the overall phylogenetic network structures were changed, thereby suggesting a potential change in the organization of intestinal microbial communities (Faust and Raes, 2012). This change may result in the potential switching of roles of intestinal microbial species and ecological functions, which may influence therapeutic effects of chemotherapy.

The identified modules within the networks probably arise from microbes–microbes interactions in response to shared niches in the intestine. The links in the ecological network could explain the co-occurrence or co-exclusion of two nodes caused by the species performing similar or complementary and contrary or exclusive functions (Zhou et al., 2011). This study results showed that the links of modules in T5 were distinctly decreased than that in T0, but still higher than that in H (Figure 1), indicating that interspecies interactions within the ecological network were changed by chemotherapy, and the more complex and compact of modules were in CRC patients. Positive interactions often indicate that nodes complement or cooperate with one another, while negative interactions signify predation or competition between the taxa. In a relatively healthy intestine, cooperation probably linked with stability was found to be the primary interaction among symbiotic intestinal communities (Jain and Krishna, 2001; Schluter and Foster, 2012). Our results showed that more negative correlations were in CRC patients (Figure 1). The previous study experimentally demonstrated that external disturbance promoted microbial species-species competition (Cyrille et al., 2010). After five stages of therapy, the negative links decreased distinctly in T5 than in T0, suggesting that chemotherapy could have key effects on species-species interactions of intestinal microbiota.

Identifying the key OTUs in a community is a challenge, because of the uncultured status and high diversity of microbes (Faust and Raes, 2012). In this study, pMENs analysis provided information on keystone OTUs that were most important to the structures and functions of microbial ecosystem in CRC patients during the different treatment stage. The keystone OTUs included that the module hubs (those highly linked to numerous OTUs in their own modules), and connectors (those highly connected to several modules). Module hubs and connectors have been proposed to be keystone taxa because losing them would result in the deterioration of the entire network (Deng et al., 2012). Topologically, different OTUs play individual roles in the ecological networks (Guimerà et al., 2007). The topological roles of the taxa were different and the keystone taxa were changed for CRC patients during the chemotherapy. We found that these keystone taxa (OTU787, OTU167, OTU8, OTU238, OTU9, OTU2, OTU847, OTU488, and OTU16) were not significantly correlated with types of chemotherapy, except for the OTU787 (*P* = 0.003), which was from the genus *Klebsiella* (Supplementary Table S6). One important reason was that most CRC patients used the same XELOX as the treatment protocols (Supplementary Table S1). The OTU167, OTU8, and OTU9 were classified into module hubs or networks hubs in T0, T1, T2, and T3, which were significantly correlated with tumor markers (*P* < 0.05, Supplementary Table S6). Therefore, these OTUs were probably extremely influenced by chemotherapy, which also may influence the chemotherapy efficacy. However, intestinal microbiota exerts both direct and indirect effects on chemotherapy efficacy by a large suite of ways. Not only are the intestinal microbiota niche-specific, but their ecology is also dynamic (Alexander et al., 2017). For example, intestinal structural ecology of CRC patients at old age might be perturbed by the exposure to a lifetime of environmental modifiers. The chemotherapy may create a state of dysbiosis, exaggerate the influence of deleterious bacteria, reduce efficacy, and further exacerbate any dysbiotic state rather than correct it (Claesson et al., 2011). Therefore, the actual roles of keystone taxa must be elucidated by co-culture experiments or more biological replicates in the further work.

It is very important to detect the interactive relationships among microbial communities across relatively large numbers of samples using network analysis (Barberán et al., 2012). We could not scale the results to all the conditions with only 22 samples, however, constructing a pMEN is important to determine the potential interactions among different microbes. The study results would provide a better understanding of the responses of intestinal microbial communities to chemotherapy. Additional larger sampling efforts are required to reduce the variables, such as age, sex, types of chemotherapy, antibiotic, and probiotics usage, to obtain more fundamental insight into intestinal microbial ecological networks in complex intestinal environment.

## Conclusion

An ecological molecular network is considered an effective way to systematically evaluate intestinal microbial homeostasis and provides novel insights into the intestinal microbiota for CRC patients in response to chemotherapy. In this study, we concluded that the intestinal microbiota of CRC patients were changed greatly under the chemotherapy based on the ecological molecular network analysis. These changes may influence the therapeutic effects of chemotherapy. However, further studies are needed to provide for more evidence to support this idea.

## Author Contributions

JC analyzed the data and wrote the manuscript. DL, NZ, and MJ collected samples and analyzed the data. JZ, CZ, TL, KL, HH, and XZ reviewed and revised the manuscript. All the authors were involved in the design of the study, interpreted the data, and read and approved the final manuscript.

## Conflict of Interest Statement

The authors declare that the research was conducted in the absence of any commercial or financial relationships that could be construed as a potential conflict of interest.

## Footnotes

1 http://ieg2.ou.edu/MENA/
