# Supplementary material for: Chemotherapy Alters the Phylogenetic Molecular Ecological Networks of Intestinal Microbial Communities
Source: Front Microbiol. 2019 May 7;10:1008. doi: 10.3389/fmicb.2019.01008 (PMC6524687; doi:10.3389/fmicb.2019.01008)
Supplement: Supplementary file 1 [file Table_1.docx]

Table S1 Clinicopathological variables in colorectal cancer patients

| CRC patients | Tumor location | Surgery | TNM^a^ stage | Grade of Differentiation | Treatment  (Five stages) |
| --- | --- | --- | --- | --- | --- |
| T1 | Rectum | Yes | III | Moderate-low | XELOX |
| T2 | Rectum | Yes | III | Moderate | XELOX |
| T3 | Colon | Yes | III | Moderate | XELOX |
| T4 | Rectum | Yes | IV | Moderate-low | XELOX |
| T5 | Colon | No | IV | Moderate | B |
| T6 | Rectum+colon | Yes | III | Moderate | SOX |
| T7 | Rectum | Yes | III | Moderate | SOX |
| T8 | Rectum | Yes | III | low | C |
| T9 | Rectum | Yes | III | Moderate | XELOX+radiotherapy |
| T10 | Rectum | Yes | III | Moderate | SOX |
| T11 | Rectum | Yes | III | High | XELOX |
| T12 | Rectum | Yes | III | Moderate | XELOX |
| T13 | Colon | Yes | IV | Moderate | XELOX |
| T14 | Colon | Yes | III | Moderate | XELOX |
| T15 | Rectum | Yes | IV | Moderate | XELOX |
| T16 | Rectum | Yes | II | Moderate | XELOX+radiotherapy |
| T17 | Colon | Yes | III | Moderate | XELOX |
| T18 | Rectum | Yes | III | Moderate | XELOX+SOX |
| T19 | Rectum | Yes | III | Moderate | XELOX |
| T20 | Colon | Yes | III | Moderate | XELOX |
| T21 | Colon | Yes | IV | Moderate-low | XELOX |
| T22 | Colon | Yes | II | Moderate | XELOX |

^a^ NCCN staging; XELOX: Oxaliplatin+Capecitabine; B: Oxaliplatin+Capecitabine for the first stage, Bevacizumab+Oxaliplatin+Capecitabine for the second, third and fourth stage, Bevacizumab+Irinotecan+Capecitabine for the fifth stage; SOX: Oxaliplatin+Tegafur, Gimeracil and Oteracil Porassium Capsules; C: XELOX for the first stage, Oxaliplatin+egafur, Gimeracil and Oteracil Porassium Capsules for the second and third stage; Irinotecan+Raltitrexed for the fourth stage; Irinotecan+Raltitrexed+[cetuximab](javascript:;) for the fifth stage; XELOX+radiotherapy: XELOX for the first four stages, radiotherapy for the fifth stage; XELOX+SOX: XELOX for the first two stages, SOX for the last three stages.

Table S2 Number of modules (with nodes＞4) present in healthy individuals and colorectal cancer patients networks.

| Terms | Healthy individuals | Colorectal cancer patients | | | | | |
| --- | --- | --- | --- | --- | --- | --- | --- |
|  | H | T0 | T1 | T2 | T3 | T4 | T5 |
| Sub-module | 8 | 6 | 6 | 6 | 6 | 7 | 7 |
| Nodes | 81 | 95 | 97 | 97 | 76 | 93 | 94 |
| Links | 98 | 236 | 223 | 214 | 171 | 203 | 226 |

Table S3 The module hubs of intestinal microbiota in CRC patients and healthy individuals

| Sample | Number | Module hubs |
| --- | --- | --- |
| H | 1 | OTU736(p__Bacteroidetes;c__Bacteroidia;o__Bacteroidales;f__Prevotellaceae;g__Prevotella) |
| T0 | 1 | OTU787(p__Proteobacteria;c__Gammaproteobacteria;o__Enterobacteriales;f__Enterobacteriaceae;g__Klebsiella) |
| T1 | 1 | OTU167(p__Fusobacteria;c__Fusobacteriia;o__Fusobacteriales;f__Fusobacteriaceae;g__Fusobacterium) |
| T2 | 2 | OTU8(p__Bacteroidetes;c__Bacteroidia;o__Bacteroidales;f__Bacteroidaceae;g__Bacteroides)  OTU238(p__Actinobacteria;c__Actinobacteria;o__Coriobacteriales;f__Coriobacteriaceae;g__Atopobium) |
| T3 | 1 | OTU9(p__Firmicutes;c__Clostridia;o__Clostridiales;f__Ruminococcaceae;g__Faecalibacterium) |
| T4 | 1 | OTU2(p__Bacteroidetes;c__Bacteroidia;o__Bacteroidales;f__Bacteroidaceae;g__Bacteroides) |
| T5 | 1 | OTU9(p__Firmicutes;c__Clostridia;o__Clostridiales;f__Ruminococcaceae;g__Faecalibacterium) |

Table S4 The connectors of intestinal microbiota in CRC patients and healthy individuals

| Sample | No. | **Connectors** |
| --- | --- | --- |
| H | 3 | OTU288(p__Firmicutes;c__Clostridia;o__Clostridiales;f__Lachnospiraceae;g__Lachnospiracea_incertae_sedis)  OTU32(p__Firmicutes;c__Clostridia;o__Clostridiales;f__Lachnospiraceae;g__Coprococcus)  OTU374(p__Firmicutes;c__Clostridia;o__Clostridiales;f__Ruminococcaceae) |
| T0 | 13 | OTU884(p__Bacteroidetes;c__Bacteroidia;o__Bacteroidales;f__Bacteroidaceae;g__Bacteroides)  OTU144(p__Firmicutes;c__Clostridia;o__Clostridiales;f__Lachnospiraceae;g__Lachnospiracea_incertae_sedis)  OTU95(p__Firmicutes;c__Clostridia;o__Clostridiales;f__Ruminococcaceae)  OTU86(p__Bacteroidetes;c__Bacteroidia;o__Bacteroidales;f__Rikenellaceae;g__Alistipes)  OTU61(p__Proteobacteria;c__Betaproteobacteria;o__Burkholderiales;f__Sutterellaceae;g__Parasutterella)  OTU238(p__Actinobacteria;c__Actinobacteria;o__Coriobacteriales;f__Coriobacteriaceae;g__Atopobium)  OTU199(p__Bacteroidetes;c__Bacteroidia;o__Bacteroidales;f__Bacteroidaceae;g__Bacteroides)  OTU24(p__Bacteroidetes;c__Bacteroidia;o__Bacteroidales;f__Rikenellaceae;g__Alistipes)  OTU262(p__Firmicutes;c__Clostridia;o__Clostridiales;f__Lachnospiraceae;g__Blautia)  OTU488(p__Proteobacteria;c__Gammaproteobacteria;o__Enterobacteriales;f__Enterobacteriaceae)  OTU735(p__Firmicutes;c__Clostridia;o__Clostridiales;f__Lachnospiraceae;g__Clostridium XlVa)  OTU784(p__Firmicutes;c__Clostridia;o__Clostridiales;f__Ruminococcaceae;g__Oscillibacter)  OTU134(p__Firmicutes;c__Bacilli;o__Lactobacillales;f__Carnobacteriaceae;g__Granulicatella)  OTU865(p__Firmicutes;c__Clostridia;o__Clostridiales;f__Lachnospiraceae;g__Clostridium XlVa)  OTU776(p__Firmicutes;c__Clostridia;o__Clostridiales;f__Lachnospiraceae;g__Clostridium XlVa)  OTU404(p__Firmicutes;c__Clostridia;o__Clostridiales;f__Ruminococcaceae)  OTU106(p__Firmicutes;c__Clostridia;o__Clostridiales;f__Lachnospiraceae;g__Clostridium XlVa)  OTU518(p__Firmicutes;c__Clostridia;o__Clostridiales;f__Lachnospiraceae;g__Clostridium XlVa) |
| T1 | 13 | OTU94(p__Firmicutes;c__Clostridia;o__Clostridiales;f__Lachnospiraceae)  OTU39(p__Firmicutes;c__Clostridia;o__Clostridiales;f__Lachnospiraceae)  OTU8(p__Bacteroidetes;c__Bacteroidia;o__Bacteroidales;f__Bacteroidaceae;g__Bacteroides)  OTU388(p__Firmicutes;c__Clostridia;o__Clostridiales;f__Lachnospiraceae)  OTU195(p__Firmicutes;c__Clostridia;o__Clostridiales;f__Lachnospiraceae;g__Lachnospiracea_incertae_sedis)  OTU162(p__Firmicutes;c__Clostridia;o__Clostridiales;f__Ruminococcaceae)  OTU231(p__Firmicutes;c__Erysipelotrichia;o__Erysipelotrichales;f__Erysipelotrichaceae;g__Clostridium XVIII)  OTU4(p__Firmicutes;c__Negativicutes;o__Selenomonadales;f__Acidaminococcaceae;g__Phascolarctobacterium)  OTU670(p__Firmicutes;c__Clostridia;o__Clostridiales;f__Lachnospiraceae)  OTU856(p__Firmicutes;c__Clostridia;o__Clostridiales;f__Ruminococcaceae;g__Oscillibacter)  OTU106(p__Firmicutes;c__Clostridia;o__Clostridiales;f__Lachnospiraceae;g__Clostridium XlVa)  OTU100(p__Bacteroidetes;c__Bacteroidia;o__Bacteroidales;f__Porphyromonadaceae;g__Odoribacter)  OTU47(p__Firmicutes;c__Clostridia;o__Clostridiales;f__Ruminococcaceae;g__Oscillibacter) |
| T2 | 13 | OTU884(p__Bacteroidetes;c__Bacteroidia;o__Bacteroidales;f__Bacteroidaceae;g__Bacteroides)  OTU90(p__Firmicutes;c__Clostridia;o__Clostridiales;f__Lachnospiraceae;g__Anaerostipes)  OTU39(p__Firmicutes;c__Clostridia;o__Clostridiales;f__Lachnospiraceae)  OTU654(p__Firmicutes;c__Clostridia;o__Clostridiales;f__Ruminococcaceae;g__Faecalibacterium)  OTU80(p__Firmicutes;c__Clostridia;o__Clostridiales;f__Lachnospiraceae;g__Clostridium XlVb)  OTU195(p__Firmicutes;c__Clostridia;o__Clostridiales;f__Lachnospiraceae;g__Lachnospiracea_incertae_sedis)  OTU659(p__Firmicutes;c__Clostridia;o__Clostridiales;f__Lachnospiraceae;g__Blautia)  OTU58(p__Firmicutes;c__Clostridia;o__Clostridiales)  OTU670(p__Firmicutes;c__Clostridia;o__Clostridiales;f__Lachnospiraceae)  OTU11(p__Firmicutes;c__Clostridia;o__Clostridiales;f__Ruminococcaceae;g__Gemmiger)  OTU856(p__Firmicutes;c__Clostridia;o__Clostridiales;f__Ruminococcaceae;g__Oscillibacter)  OTU106(p__Firmicutes;c__Clostridia;o__Clostridiales;f__Lachnospiraceae;g__Clostridium XlVa)  OTU47(p__Firmicutes;c__Clostridia;o__Clostridiales;f__Ruminococcaceae;g__Oscillibacter) |
| T3 | 20 | OTU144(p__Firmicutes;c__Clostridia;o__Clostridiales;f__Lachnospiraceae;g__Lachnospiracea_incertae_sedis)  OTU92(p__Firmicutes;c__Clostridia;o__Clostridiales;f__Ruminococcaceae;g__Oscillibacter)  OTU95(p__Firmicutes;c__Clostridia;o__Clostridiales;f__Ruminococcaceae)  OTU39(p__Firmicutes;c__Clostridia;o__Clostridiales;f__Lachnospiraceae)  OTU388(p__Firmicutes;c__Clostridia;o__Clostridiales;f__Lachnospiraceae)  OTU86(p__Bacteroidetes;c__Bacteroidia;o__Bacteroidales;f__Rikenellaceae;g__Alistipes)  OTU80(p__Firmicutes;c__Clostridia;o__Clostridiales;f__Lachnospiraceae;g__Clostridium XlVb)  OTU232(p__Actinobacteria;c__Actinobacteria;o__Actinomycetales;f__Micrococcaceae;g__Rothia)  OTU767(p__Firmicutes;c__Clostridia;o__Clostridiales;f__Lachnospiraceae;g__Roseburia)  OTU658(p__Firmicutes;c__Clostridia;o__Clostridiales;f__Lachnospiraceae;g__Ruminococcus2)  OTU50(p__Firmicutes;c__Clostridia;o__Clostridiales;f__Ruminococcaceae;g__Ruminococcus)  OTU352(p__Firmicutes;c__Clostridia;o__Clostridiales;f__Lachnospiraceae)  OTU174(p__Firmicutes;c__Bacilli;o__Bacillales;f__Bacillales_Incertae Sedis XI;g__Gemella)  OTU19(p__Bacteroidetes;c__Bacteroidia;o__Bacteroidales;f__Porphyromonadaceae;g__Parabacteroides)  OTU15(p__Firmicutes;c__Clostridia;o__Clostridiales;f__Lachnospiraceae;g__Clostridium XlVa)  OTU821(p__Firmicutes;c__Clostridia;o__Clostridiales;f__Ruminococcaceae;g__Ruminococcus)  OTU106(p__Firmicutes;c__Clostridia;o__Clostridiales;f__Lachnospiraceae;g__Clostridium XlVa)  OTU105(p__Firmicutes)  OTU47(p__Firmicutes;c__Clostridia;o__Clostridiales;f__Ruminococcaceae;g__Oscillibacter)  OTU211(p__Firmicutes;c__Clostridia;o__Clostridiales;f__Lachnospiraceae) |
| T4 | 10 | OTU149(p__Candidatus Saccharibacteria;g__Saccharibacteria_genera_incertae_sedis)  OTU92(p__Firmicutes;c__Clostridia;o__Clostridiales;f__Ruminococcaceae;g__Oscillibacter)  OTU1010(p__Bacteroidetes;c__Bacteroidia;o__Bacteroidales;f__Bacteroidaceae;g__Bacteroides)  OTU86(p__Bacteroidetes;c__Bacteroidia;o__Bacteroidales;f__Rikenellaceae;g__Alistipes)  OTU735(p__Firmicutes;c__Clostridia;o__Clostridiales;f__Lachnospiraceae;g__Clostridium XlVa)  OTU352(p__Firmicutes;c__Clostridia;o__Clostridiales;f__Lachnospiraceae)  OTU670(p__Firmicutes;c__Clostridia;o__Clostridiales;f__Lachnospiraceae)  OTU174(p__Firmicutes;c__Bacilli;o__Bacillales;f__Bacillales_Incertae Sedis XI;g__Gemella)  OTU776(p__Firmicutes;c__Clostridia;o__Clostridiales;f__Lachnospiraceae;g__Clostridium XlVa)  OTU856(p__Firmicutes;c__Clostridia;o__Clostridiales;f__Ruminococcaceae;g__Oscillibacter) |
| T5 | 16 | OTU143(p__Proteobacteria;c__Gammaproteobacteria;o__Enterobacteriales;f__Enterobacteriaceae)  OTU97(p__Firmicutes;c__Clostridia;o__Clostridiales;f__Lachnospiraceae;g__Coprococcus)  OTU39(p__Firmicutes;c__Clostridia;o__Clostridiales;f__Lachnospiraceae)  OTU277(p__Firmicutes;c__Clostridia;o__Clostridiales;f__Ruminococcaceae)  OTU82(p__Bacteroidetes;c__Bacteroidia;o__Bacteroidales;f__Bacteroidaceae;g__Bacteroides)  OTU62(p__Firmicutes;c__Clostridia;o__Clostridiales;f__Lachnospiraceae;g__Blautia)  OTU488(p__Proteobacteria;c__Gammaproteobacteria;o__Enterobacteriales;f__Enterobacteriaceae)  OTU50(p__Firmicutes;c__Clostridia;o__Clostridiales;f__Ruminococcaceae;g__Ruminococcus)  OTU178(p__Candidatus Saccharibacteria;g__Saccharibacteria_genera_incertae_sedis)  OTU14(p__Firmicutes;c__Clostridia;o__Clostridiales;f__Ruminococcaceae)  OTU821(p__Firmicutes;c__Clostridia;o__Clostridiales;f__Ruminococcaceae;g__Ruminococcus)  OTU107(p__Firmicutes;c__Clostridia;o__Clostridiales;f__Ruminococcaceae)  OTU106(p__Firmicutes;c__Clostridia;o__Clostridiales;f__Lachnospiraceae;g__Clostridium XlVa)  OTU105(p__Firmicutes)  OTU45(p__Bacteroidetes;c__Bacteroidia;o__Bacteroidales;f__Rikenellaceae;g__Alistipes)  OTU47(p__Firmicutes;c__Clostridia;o__Clostridiales;f__Ruminococcaceae;g__Oscillibacter) |

Table S5 The network hubs of intestinal microbiota in CRC patients and healthy individuals

| **Sample** | **Number** | **Network hubs** |
| --- | --- | --- |
| **H** | 0 | **/** |
| **T0** | 1 | OTU167(p__Fusobacteria;c__Fusobacteriia;o__Fusobacteriales;f__Fusobacteriaceae;g__Fusobacterium) |
| **T1** | 2 | OTU488(p__Proteobacteria;c__Gammaproteobacteria;o__Enterobacteriales;f__Enterobacteriaceae)  OTU16(p__Firmicutes;c__Clostridia;o__Clostridiales;f__Clostridiaceae 1;g__Clostridium sensu stricto) |
| **T2** | 3 | OTU787(p__Proteobacteria;c__Gammaproteobacteria;o__Enterobacteriales;f__Enterobacteriaceae;g__Klebsiella)  OTU488(p__Proteobacteria;c__Gammaproteobacteria;o__Enterobacteriales;f__Enterobacteriaceae)  OTU16(p__Firmicutes;c__Clostridia;o__Clostridiales;f__Clostridiaceae 1;g__Clostridium sensu stricto) |
| **T3** | 1 | OTU238(p__Actinobacteria;c__Actinobacteria;o__Coriobacteriales;f__Coriobacteriaceae;g__Atopobium) |
| **T4** | 0 | **/** |
| **T5** | 1 | OTU847(p__Proteobacteria;c__Gammaproteobacteria;o__Enterobacteriales;f__Enterobacteriaceae;g__Escherichia/Shigella) |

Table S6 The Spearman correlation of key OTUs and tumor markers and types of chemotherapy.

| Taxa | CA242(r/*P*) | CEA(r/*P*) | CA199(r/*P*) | CA724(r/*P*) | Types of chemotherapy |
| --- | --- | --- | --- | --- | --- |
| OTU787 | 0.008/0.949 | 0.065/0.596 | 0.015/0.903 | 0.126/0.306 | **0.292/0.003** |
| OTU167 | 0.038/0.779 | **0.418/0.001** | 0.067/0.613 | 0.03/0.82 | 0.154/0.124 |
| OTU8 | 0.021/0.444 | **0.220/0.027** | 0.01/0.923 | **0.279/0.005** | 0.091/0.365 |
| OTU238 | 0.105/0.444 | 0.171/0.208 | 0.042/0.758 | 0.107/0.431 | -0.100/0.318 |
| OTU9 | **0.198/0.046** | **0.258/0.008** | 0.126/0.199 | 0.03/0.76 | 0.129/0.198 |
| OTU2 | 0.039/0.689 | 0.025/0.793 | 0.151/0.113 | 0.097/0.314 | 0.091/0.367 |
| OTU847 | 0.118/0.445 | 0.181/0.225 | 0.275/0.062 | 0.097/0.522 | 0.050/0.618 |
| OTU488 | 0.013/0.921 | 0.131/0.028 | 0.031/0.799 | 0.208/0.088 | 0.014/0.890 |
| OTU16 | 0.093/0.596 | 0.137/0.4 | 0.168/0.293 | 0.172/0.301 | 0.115/0.251 |

“r” means the Spearman’s correlation coefficient; “*P*” means the significant results.

OTU787(p__Proteobacteria;c__Gammaproteobacteria;o__Enterobacteriales;f__Enterobacteriaceae;g__Klebsiella); OTU167(p__Fusobacteria;c__Fusobacteriia;o__Fusobacteriales;f__Fusobacteriaceae;g__Fusobacterium); OTU8(p__Bacteroidetes;c__Bacteroidia;o__Bacteroidales;f__Bacteroidaceae;g__Bacteroides); OTU238(p__Actinobacteria;c__Actinobacteria;o__Coriobacteriales;f__Coriobacteriaceae;g__Atopobium); OTU9(p__Firmicutes;c__Clostridia;o__Clostridiales;f__Ruminococcaceae;g__Faecalibacterium); OTU2(p__Bacteroidetes;c__Bacteroidia;o__Bacteroidales;f__Bacteroidaceae;g__Bacteroides); OTU847(p__Proteobacteria;c__Gammaproteobacteria;o__Enterobacteriales;f__Enterobacteriaceae;g__Escherichia/Shigella); OTU488(p__Proteobacteria;c__Gammaproteobacteria;o__Enterobacteriales;f__Enterobacteriaceae); OTU16(p__Firmicutes;c__Clostridia;o__Clostridiales;f__Clostridiaceae 1;g__Clostridium sensu stricto)
