# Supplementary material for: Chemotherapy Alters the Phylogenetic Molecular Ecological Networks of Intestinal Microbial Communities
Source: Front Microbiol. 2019 May 7;10:1008. doi: 10.3389/fmicb.2019.01008 (PMC6524687; doi:10.3389/fmicb.2019.01008)
Supplement: Supplementary file 2 [file Image_1.pdf]

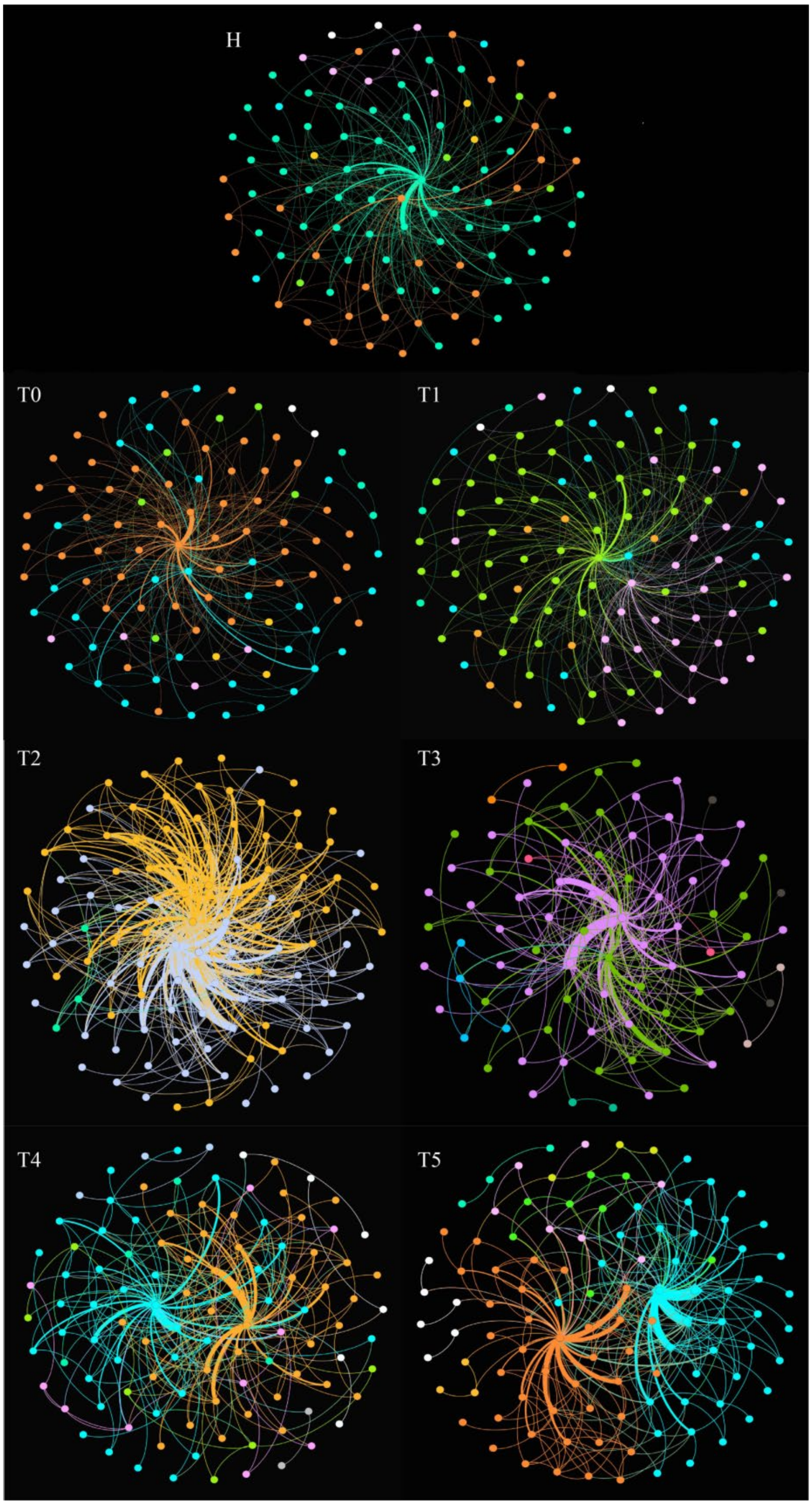

Figure S1 Overview of the networks in healthy individuals and colorectal cancer patients in response to the five stages of chemotherapy. The modules are randomly coloured at each stage of chemotherapy, and nodes in modules with a relative abundance < 1% are in white. Networks represent random matrix theory co-occurrence models derived from more than 19 biological replicates at each stage, where nodes represent OTUs and links between the nodes indicate the significant correlation.

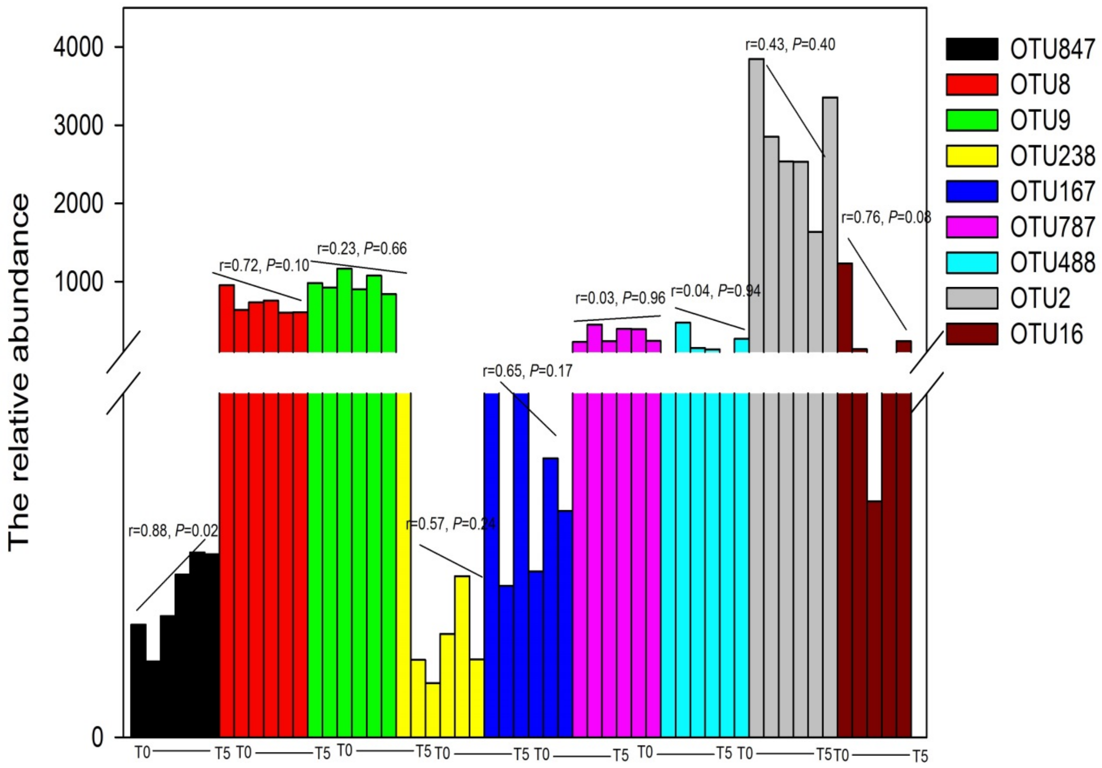

Figure S2 Changes of the keystone taxa in response to chemotherapy.
